# Reliability of Baropodometry on the Evaluation of Plantar Load Distribution: A Transversal Study

**DOI:** 10.1155/2017/5925137

**Published:** 2017-03-02

**Authors:** Daniel Baumfeld, Tiago Baumfeld, Romário Lopes da Rocha, Benjamim Macedo, Fernando Raduan, Roberto Zambelli, Thiago Alexandre Alves Silva, Caio Nery

**Affiliations:** ^1^UFMG, Belo Horizonte, MG, Brazil; ^2^HC-UFMG, Belo Horizonte, MG, Brazil; ^3^Baleia Hospital, Belo Horizonte, MG, Brazil; ^4^Felício Rocho Hospital, Belo Horizonte, MG, Brazil; ^5^UNIFESP, São Paulo, SP, Brazil; ^6^Mater Dei Hospital, Belo Horizonte, MG, Brazil; ^7^Madre Teresa Hospital, Belo Horizonte, MG, Brazil

## Abstract

*Introduction*. Baropodometry is used to measure the load distribution on feet during rest and walking. The aim of this study was to evaluate changes in plantar foot pressures distribution due to period of working and due to stretching exercises of the posterior muscular chain.* Methods*. In this transversal study, all participants were submitted to baropodometric evaluation at two different times: before and after the working period and before and after stretching the muscles of the posterior chain.* Results*. We analyzed a total of 54 feet of 27 participants. After the working period, there was an average increase in the forefoot pressure of 0.16 Kgf/cm^2^ and an average decrease in the hindfoot pressure of 0.17 Kgf/cm^2^. After stretching the posterior muscular chain, the average increase in the forefoot pressure was 0.56 Kgf/cm^2^ and the hindfoot average pressure decrease was 0.56 Kgf/cm^2^. These changes were not statistically significant.* Discussion*. It was reported that the strength of the Achilles tendon generates greater forefoot load transferred from the hindfoot. In our study, no significant variation in the distribution of plantar pressure was observed. It can be inferred that baropodometry was a reliable instrument to determine the plantar pressure, regardless of the tension of the posterior chain muscles.

## 1. Introduction

The human foot acts as a base for support and propulsion in gait, ensuring effectiveness in load transfer during the entire gait cycle [1]. Proper biomechanics of the foot is responsible for maintaining the posture and symmetrical distribution of the plantar pressure [1–3]. The study of plantar pressures has been well discussed in several papers, but its real clinical applicability has not yet been determined and validated [4]. With its two modalities, static or dynamic, baropodometry is the main tool for the study of plantar pressures distribution [5].

The baropodometer is an advanced force platform, used for the analysis of plantar pressure areas applied by the body in both motion and static position. It uses appropriate software to produce images similar to a podoscope. This technique provides data with a high diagnostic value, which are printed in graphs. It provides direct and indirect information about the position of the patient in the standing position, dynamic gait analysis, distribution of loads during walking, peak pressure and contact time with the ground, and detection of areas in risk on foot and helps in the production of orthotic insoles, on the detection of biomechanical abnormalities of the foot, pelvis, and spine. With baropodometry, different authors suggest the treatment of different postural problems using appropriate stretching and/or use of different shoe insoles [6].

Several scientific evidences suggests that the contracture of the posterior tendons and calf muscle bellies increases the load transfer from the hindfoot to the forefoot, changing the load distribution on the plantar surface, which could influence on the emergence of calluses, metatarsalgia, lesser toes deformities, and skin ulcers [2]. In a randomized study with patients with diabetic forefoot ulcers, surgical lengthening of the Achilles tendon decreased the number of reulceration, due to decreased pressure on the forefoot [7, 8].

The purpose of this study was to evaluate if there are any changes in plantar foot pressures distribution due to a regular period of working and due to stretching exercises of the posterior muscular chain of the lower extremities in normal subjects.

## 2. Material and Methods

This is a transversal study settled in March of 2013. All nursing professionals of our Hospital were invited to participate in this study. After excluding those who had previous orthopedic and traumatic pathologies and/or foot and ankle complains, forty hospital nursing professionals signed a free and informed consent to be included in this study. All participants were female (average age of 35 years/average weight of 59 kg/average height of 1,69 m) and worked in the hospital in a 12-hour shift.

All participants answered a questionnaire with anthropometric and medical data (weight, height, and size of the feet, other morbidities) and were submitted to static baropodometric evaluation at two different times (four measurements). In the first part of the study, they were analyzed before the working period, between 07:00 and 08:00 a.m., and after the end of a journey, between 07:00 and 08:00 p.m. In the second part, the same individuals were assessed during the morning before and after stretching the muscles of the posterior chain for 10 minutes. All stretching exercises were guided and supervised by an experienced physiotherapist and a Foot and Ankle Surgeon (DB).

**Figure 1:**
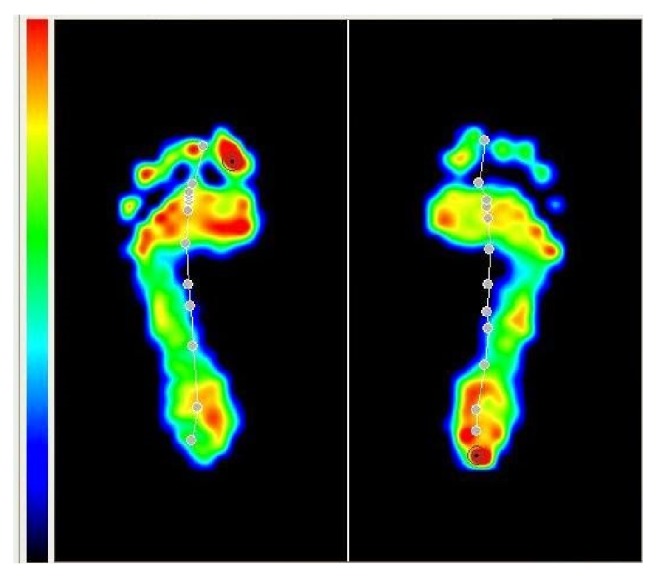
Example of a baropodometric measurement.

For the baropodometric evaluation, all individuals remained in a standing, static, bipedal position with the arms pending along the body over the platform with their eyes open mirrored to a fixed point on the wall of the examination room. They stayed on the platform for an average of 60 seconds to perform the calibration and measurements. All subjects were evaluated in the same baropodometer (EPS R-1-KINETEC®). The forefoot was assumed as the foot part anterior to the gravity center and the hindfoot as the part posterior to the center of gravity registered on the device (Figure 1).

Participants who did not perform both measurements in each step were excluded from this study. Twenty-seven patients concluded both steps of the study.

Statistical analyses were performed using Minitab 16 and Excel 2013, with the Anderson-Darling (as the normality test) and the paired *T*-test (for crossing data).

This study was conducted with the human subjects' understanding and consent. Our hospital Ethical Committee has approved this work.

## 3. Results

Table 1 describes the average pressure data in Kgf/cm^2^ of the 27 patients (54 feet) before and after the working period.

Table 2 describes the average pressure data in Kgf/cm^2^ of the 27 patients (54 feet) before and after stretching the posterior muscular chain.

Table 3 shows the differences of pressure (Kgf/cm^2^) found before and after the working period. Statistical analysis demonstrated that both forefoot and hindfoot pressures did not change with a single day of working (*p* = 0,51 and *p* = 0,86, resp.).

Table 4 shows the differences of pressure (Kgf/cm^2^) found before and after stretching the posterior chain. Statistical analysis demonstrated that both forefoot and hindfoot pressures did not change (*p* = 0,12 and *p* = 0,9, resp.).

## 4. **Discussion**

The plantar pressure measurement is not widely used in clinical practice. Even in research settings, indications and use are limited. Nevertheless, according to Putti et al. [9] its potential is highly recognized in atypical Parkinsonism diagnosis [10], diabetics plantar load distribution [11], and evaluation of postural alignment on foot pressure [12].

One of the problems of using a baropodometer is its low reproducibility, with interference of many factors that could product bias, such as the sensor technology, spatial resolution of the software, pressure distribution analysis, and calibration procedures (known as the “Achilles heel”). The baropodometry standardization of measurements and calibration still need further studies and advances [13–15].

Regarding the type of apparatus used, it can be divided into three types: platform, similar to that used in this study, where the measurement is performed between the foot and the ground; insole type, with measurement between the foot and footwear; gait track type, which works as an extended platform, which is better for gait study [13, 15]. In general, the image produced is similar to the one that appears in a podoscope, with the only difference being that the baropodometer expresses different pressures within a spectrum of colors depending of pressures [13, 15].

Cadaveric studies using the baropodometer showed that increased strength in the Achilles tendon generates greater pressure transfer from the hindfoot to the forefoot [16]. According to Armstrong et al. [17], lengthening the Achilles tendon decreases tension in the triceps complex, with decreased plantar peak pressure and reduction in the number of ulcers in diabetic patients. Likewise, Aronow [18] demonstrated that the tension in the Achilles alters the distribution of load in the foot with increased forefoot and midfoot pressure and decreased hindfoot pressure. In a randomized study of diabetic patients, Mueller et al. [8] found a lower rate of reulceration after lengthening of the Achilles tendon, with return to the initial status after 7 months.

Stretching the lower leg musculature before training and sports is a commonly practiced routine used by both professional and recreational athletes to prevent musculoskeletal injuries. However, little evidence exists demonstrating a reduction in the incidence of injury, and many clinical recommendations are based on common sense, intuition, and tradition. There are some studies that show reduction on plantar heel pain and improvement in ankle dorsiflexion with posterior muscular chain stretching, but no evidence is found about how it changes the load distribution on plantar surface [19–21].

In our study, we found no difference between load distributions on foot before and after posterior calf muscles stretching. There are also no pressure changes after a complete working day. We attempted to assess differences in plantar pressure distribution in normal individuals before and after a regular working day as well as before and after muscular stretching, checking if dynamic daily changes in the Achilles tendon and muscles could generate any changes in load distribution on feet.

One of the weaknesses of our research was the inexistence of a control group. More studies have to be done to evaluate the influence of posterior chain shortening in load distribution on feet in the clinical setting.

## 5. **Conclusion**

Our findings suggests that neither a heavy working activity nor a session of stretching exercises of the posterior muscular chain can cause detectable alterations in the feet plantar pressure distribution in normal subjects.

## Figures and Tables

**Table 1:** Average pressure data (Kgf/cm^2^) before and after the working period.

Moment of the measurement	Foot Region	Feet (*n*)	Average	Standard deviation	Max.	Min.
Before working period	Hindfoot	54	30.85	4.26	40.81	20.43
	Forefoot	54	19.15	3.71	27.46	10.93
After working period	Hindfoot	54	31.01	4.54	41.36	19.63
	Forefoot	54	18.99	3.99	31.02	11.62

**Table 2:** Average pressure data (Kgf/cm^2^) before and after stretching the posterior muscular chain.

Moment of the measurement	Foot Region	Feet (*n*)	Average	Standard deviation	Max.	Min.
Before stretching	Hindfoot	54	29.00	6.17	40.34	15.27
	Forefoot	54	21.00	5.58	34.30	8.20
After stretching	Hindfoot	54	28.44	5.08	38.13	16.60
	Forefoot	54	21.56	4.76	31.18	10.23

**Table 3:** Differences of pressure (Kgf/cm^2^) before and after the working period.

Foot site	*N*	Average	Standard deviation	Max.	Min.
Hindfoot	54	0.16	4.50	8.9	−9.63
Forefoot	54	−0.17	3.73	7.41	−10.14

*p* value (hindfoot) = 0,510/*p* value (forefoot) = 0,860.

**Table 4:** Differences of pressure (Kgf/cm^2^) before and after the stretching of the posterior chain.

Foot site	*N*	Average	Standard deviation	Max.	Min.
Hindfoot	54	−0.56	4.71	10.9	−11.56
Forefoot	54	0.56	4.75	14.21	−11.33

*p* value (hindfoot) = 0,120/*p* value (forefoot) = 0,900.
